# An Integrated Approach for Finding Overlooked Genes in *Shigella*

**DOI:** 10.1371/journal.pone.0018509

**Published:** 2011-04-05

**Authors:** Junping Peng, Jian Yang, Qi Jin

**Affiliations:** State Key Laboratory for Molecular Virology and Genetic Engineering, Institute of Pathogen Biology, Chinese Academy of Medical Sciences/Peking Union Medical College, Beijing, China; Columbia University, United States of America

## Abstract

**Background:**

The completion of numerous genome sequences introduced an era of whole-genome study. However, many genes are missed during genome annotation, including small RNAs (sRNAs) and small open reading frames (sORFs). In order to improve genome annotation, we aimed to identify novel sRNAs and sORFs in *Shigella*, the principal etiologic agents of bacillary dysentery.

**Methodology/Principal Findings:**

We identified 64 sRNAs in *Shigella,* which were experimentally validated in other bacteria based on sequence conservation. We employed computer-based and tiling array-based methods to search for sRNAs, followed by RT-PCR and northern blots, to identify nine sRNAs in *Shigella flexneri* strain 301 (*Sf*301) and 256 regions containing possible sRNA genes. We found 29 candidate sORFs using bioinformatic prediction, array hybridization and RT-PCR verification. We experimentally validated 557 (57.9%) DOOR operon predictions in the chromosomes of *Sf*301 and 46 (76.7%) in virulence plasmid.We found 40 additional co-expressed gene pairs that were not predicted by DOOR.

**Conclusions/Significance:**

We provide an updated and comprehensive annotation of the *Shigella* genome. Our study increased the expected numbers of sORFs and sRNAs, which will impact on future functional genomics and proteomics studies. Our method can be used for large scale reannotation of sRNAs and sORFs in any microbe with a known genome sequence.

## Introduction

Genome sequence information has accumulated at a fast pace in recent years. The generation of whole genome sequences creates new opportunities and resources for both basic and applied research. A complete understanding of an organism's biology depends largely on the accuracy and completeness with which it is annotated. In spite of tremendous advances in gene-finding programs, we are still a long way from thorough and robust annotations for sequenced genomes. A major problem is that many genes have been overlooked, including noncoding RNAs (ncRNAs) and small open reading frames (<100 amino acids; sORFs).

There has been considerable recent interest in ncRNAs, other than ribosomal RNAs (rRNAs) and transfer RNAs (tRNAs), as important regulators in eukaryotes and prokaryotes[1], [2], [3], [4], [5]. These RNAs are collectively referred to as small RNAs (sRNAs) in bacteria where they usually regulate gene expression by pairing with other RNAs as part of RNA-protein complexes, or adopt the structures of other nucleic acids [2], [6]. sRNAs lack primary sequence common statistical signals that might be exploited by reliable detection algorithms. thus, the genome-wide annotation of sRNAs has turned out to be a more complex and demanding problem than one expected. In recent years, new bioinformatics and experimental strategies have identified a greater number of novel sRNA candidates in bacteria, including, *Escherichia coli* [7], [8], [9], [10], [11], [12], *Vibrio cholerae* [13], [14], [15], *Staphylococcus aureus* [16], *Clostridium perfringens* [17], [18], *Chlamydia trachomatis* [19], *Pseudomonas aeruginosa* [20], [21], *Bacillus subtilis* [22], [23], *Listeria monocytogenes* [24], [25], *Salmonella typhimurium* [26], [27], [28], *Streptococcus pyogenes* [29], *Streptococcus pneumoniae* [30], [31], *Mybacterium tuberculosis* [32], and many others. At present, ∼150 bacterial sRNAs have been identified by systematic screens, direct labeling and functional genetic screens[3]. However, the function of the majority of these sRNAs is still unknown. The potential role of sRNA genes in pathogenic bacterial virulence has yet to be clarified.

Bacterial genes average ∼1000 nucleotides in sequenced genomes. Annotation of sORFs is difficult, because they are “buried” in an enormous pile of short random open reading frames (ORFs), which, makes them unfavorable targets for random mutagenesis[33]. To maintain a balance between underprediction and overprediction, we usually adopt certain arbitrary cut-offs for gene prediction, such as a 100 codon minimum ORF length. This means that many sORFs are not identified, including many with important functions, such as intercellular signals, intracellular toxins, and kinase inhibitors. Systematic analysis of the prevalence of sORFs had been performed in yeast [33], [34] and *E.coli* [35], [36] and results show that numerous sORFs were overlooked in initial annotation.

*Shigella* species are Gram negative, non-sporulating, facultative anaerobes that cause bacillary dysentery, a disease which remains a major worldwide health problem. They are sub-grouped into four species: *Shigella dysenteriae*, *Shigella flexneri*, *Shigella boydii,* and *Shigella sonnei*. However, multilocus enzyme electrophoresis, multilocus sequence typing, and comparative genomic hybridization suggest that *Shigella* diverged from *E. coli* in several independent events, which means it may not constitute a separate genus [37], [38], [39], [40]. Results from several *Shigella* genome sequencing projects suggest that many sRNAs and sORFs were overlooked during initial annotation [41], [42], [43], [44], [45]. Huang *et al.* reported that the number of sRNA genes in *S. dysenteriae*, *S. flexneri*, *S. boydii,* and *S. sonnei* were 33, 40, 35, and 38, respectively [46]. However, these results were incomplete. The majority were identified in *E.coli* K12, based on conservation, meaning that sRNAs unique to *Shigella* were missed. Therefore, we performed a systematic analysis of sRNAs in *Shigella*.

No previous reports exist of global experimental approaches for sRNA and sORF identification in the *Shigella*. Here we present a combined bioinformatic and experimental approach for finding sRNAs and sORFs in *Shigella*. Our search for sRNAs contained four steps. We conducted an initial genomic screen for sRNA candidates in the *Shigella* genome using existing sRNA sequences. We then performed *de novo* prediction using RNAz, which proved to be an efficient method for detecting sRNAs [47], [48]. Our next step was to identify transcribed intergenic regions and anti-sense strands of coding sequences. We developed an orthogonal approach to *in silico* primary sequence analysis that was based on high density oligonucleotide probe arrays, which interrogated both strands of the *S. flexneri* strain 301 (*Sf*301) genome. We interrogated both strands of a genomic sequence using one array, which obtained valuable information on possible antisense gene regulation and provided the basis for a more accurate understanding of gene translation. We concluded the analysis by performing northern blots and RT-PCRs to validate our findings. We also performed bioinformatic prediction, array hybridization and RT-PCR verification for sORFs.

## Results

### Known sRNAs in *Shigella*

Only one sRNA, RnaG, is known from the virulence plasmid (VP) of *Shigella* [49]. We conducted a comparative genomics-based search for sRNAs identified in other bacteria. Based on sequence conservation, we identified 63 other sRNAs in *Shigella* which were experimentally validated (sRNAs were documented by Northern blot analysis, as shown in Table S1). Sixty were identified in *E. coli* and the remaining three were verified in the pathogens *S. typhimurium* and *P. aeruginosa*. All 63 sRNAs were encoded by chromosomal DNA, where genesize ranged from 50–500 nucleotides. We identified sRNA functional categories, including TPP riboswitch, FMN riboswitch, putative endoribonuclease, bacterial signal recognition particle RNA, tmRNA, 6S RNA, and other functions. Hfq is one of the most abundant RNA-binding proteins in bacteria. Twenty-one *Shigella* sRNAs are known to bind Hfq and are likely to act by base pairing.

### Candidate sRNAs in *Shigella*

We used the program RNAz to predict regions encoding conserved RNA secondary structure, on the basis of BLAST sequence alignments between noncoding regions of six *Shigella* genomes. We focused our attention on sequences most likely to encode sRNAs, by excluding regions containing tRNAs, rRNAs, and transposase remnants. We also excluded segments which where conserved directly adjacent to the start of flanking coding genes, *i.e*., within 40 nt. We identified the corresponding sRNAs in *S.flexneri*.

Mant sRNAs are likely to be transcribed only under specific conditions, so we increased the probability of discovery of these sRNAs with our screening approach. We performed expression profile analysis in five different conditions using a tiling array, in which we excluded repetitive regions and small untranslated regions (UTRs) from our analysis. The sixty four confirmed sRNAs, previously mentioned, were used as controls. We detected 52 sRNAs (81.3%) using RNAz and 41 (64.1%) by array analysis. We identified 35 (54.7%) by both RNAz and array analysis, and 58 (90.6%) by only one method. Earlier studies have reported the presence of *rho*-independent transcription terminators as evidence for the identification of sRNA[8]. Of the known sRNAs, 49 (76.6%) were predicted by their *rho*-independent transcription terminators. Giangrossi *et al.* recently reported RnaG, the first sRNA encoded by the VP of *S. flexneri*, which is transcribed in *cis* on the complementary strand of *icsA* and encodes an invasion protein[49]. We detected RnaG by both RNAz and tiling array analysis.

Based on the RNAz predictions and tiling array analyses, 238 and 18 regions were identified respectively as containing possible sRNAs genes (including known sRNAs) in chromosome and VP, as shown in Table S2. According to the sORF prediction, these regions did not appear to encode small peptides. We could not accurately identify the exact transcription start/end sites for candidate sRNA, because our tiling array design had overlapping probes arranged at 25 bp intervals, which does not provide single nucleotide resolution. Thus, the start and end of sRNAs in Table S1 refers to the boundaries of transcriptionally active regions of candidate sRNAs. We verified the sRNAs we detected by tiling array analysis byconducting RT-PCR and detected 165 regions in the chromosome and 18 regions in the VP.

### Identified sRNAs

**Figure 1:**
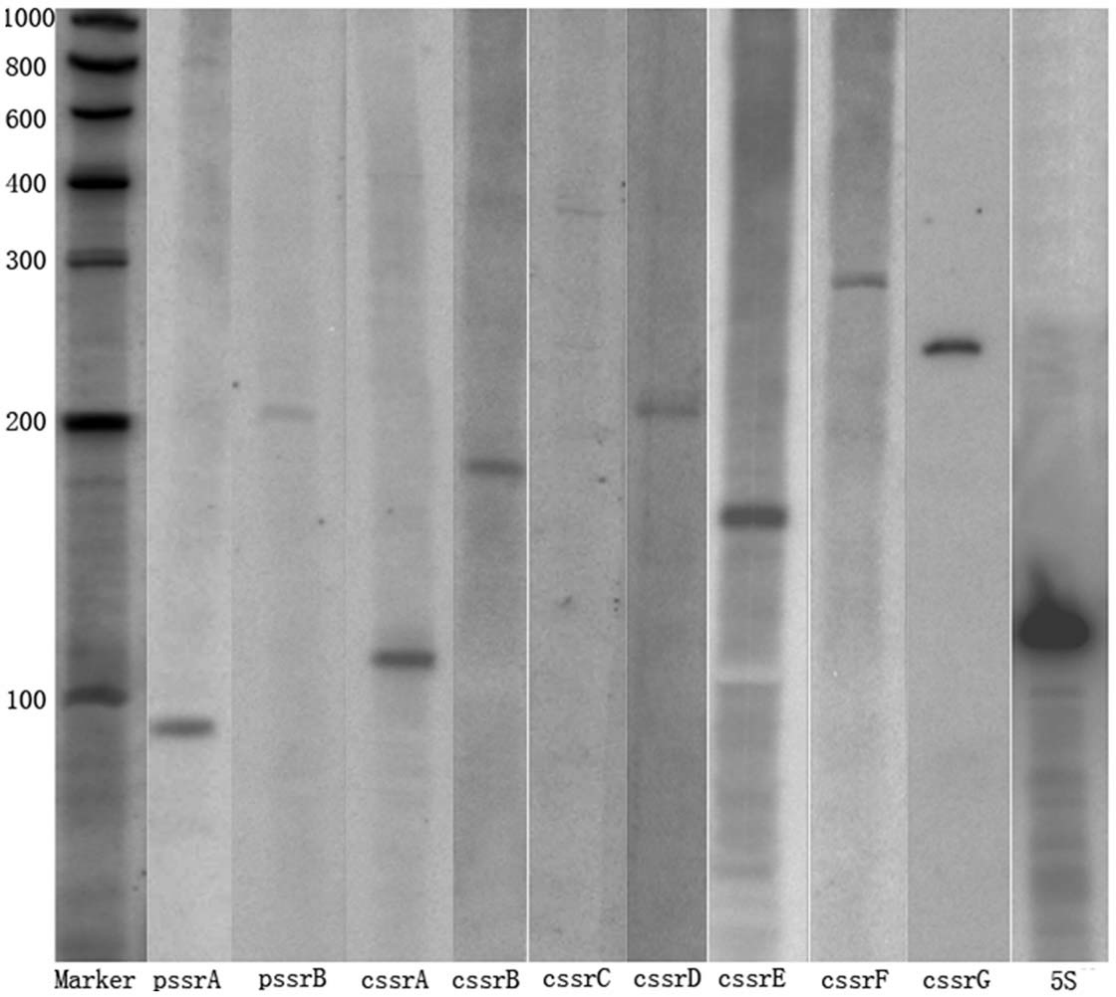
Detection of small RNAs by Northern blot analyses. Northern blots were performed with total RNA using strand-specific probes as described in Materials and Methods. The size of RNA markers is indicated on the left. 5s RNA was used as control.

We validated our sRNA predictions by northern blot analysis using 18 sequences (12 in the chromosome and 6 in the VP) detected by RNAz prediction, tiling array and *rho*-independent terminators. We successfully identified transcripts corresponding to sRNAs in nine different intergenic regions. We designated these regions as ‘pssr’ for plasmid-encoded *Shigella* small RNA, and ‘cssr’ for chromosome-encoded *Shigella* small RNA. Table 1 shows novel sRNAs which we predicted to be synthesized from their own transcription initiation sites, which were not predicted to code for proteins using the Glimmer, RBSfinder and GeneMark.hmm ORF prediction algorithms. The sRNA 3′ boundaries are based on *rho*-independent terminator predictions. Northern blot analysis indicates that the size of the sRNAs ranged from 90–340 nucleotides (Figure 1).

**Table 1:** Summary of newly confirmed sRNAs in chromosome and virulence plasmid of *Shigella flexneri* strain 301.

sRNA genes	Adjacent genes	Strand^a^	Northern size	5′ end^b^	3′end^b^	Method^c^
pssrA	CP0121/ipaJ	←→→	∼90	∼103842	103931	R/M/P
pssrB	virG/CP0183	→→→	∼200	∼152821	153020	R/M/P
cssrA	map/rpsB	←→→	∼110	∼181629	181738	R/M/P
cssrB	SF2021/SF2022	→←→	∼180	∼2046404	2046225	R/M/P
cssrC	SF2042/SF2043	←←←	∼340	∼2064237	2063898	R/M/P
cssrD	rpsP/ffh	←←←	∼200	∼2745060	2744861	R/M/P
cssrE	yggN/yggL	←←←	∼140	∼3043882	3043743	R/M/P
cssrF	dacB/yhbZ	→←←	∼290	∼3322880	3322591	R/M/P
cssrG	rbsB/rbsK	→→→	∼230	∼3946524	3946755	R/M/P

a The middle arrow represents the sRNA gene, while the flanking arrows indicate the orientation of the adjacent genes, respectively. Genes present on the strand given in the *S. flexneri* strain 301 genome database are indicated by (→), and genes present on the complementary strand are indicated by (←).

b The sRNA 3′ boundaries are from *rho*-independent terminator predictions. 5′ boundaries are calculated according to the 3′-ends and northern results.

c sRNAs were predicted based on different methods. R, RNAz prediction; M, tiling array hybridization; P: RT-PCR verification.

### Candidate sORFs

We constructed a database of predicted *Sf301* sORFs using three bioinformatics prediction software programs (data not shown). We excluded ORFs less than 25 amino acids in length and any insertion sequence-related ORFs. We performed tiling array analysis to identify overlooked sORFs in regions previously considered to be intergenic and detected 20 novel sORF candidates located within regions of the *Sf*301 chromosome and 9 in the VP. The size of these sORFs ranged from 28 to 94 codons, including start and stop codons, as shown in Table 2. We successfully verified all sORFs detected by tiling array analysis using RT-PCR. We performed BLASTX searches for functional annotation against the nonredundant protein database of the NCBI. We found that four newly identified sORFs were not annotated in any genome and four sORFs were only annotated in one *E.coli* or *Shigella* strain.

**Table 2:** Summary of candidate sORFs in chromosome and virulence plasmid of *Shigella flexneri* strain 301.

ID	Location	Length (amino acids)	Strand	Description
Chromosome				
BIO00004	15610–15401	70	−	regulatory protein mokC
BIO00051	259932–259741	64	−	hypothetical protein
BIO00126	511407–511556	50	+	putative small toxic membrane polypeptide
BIO00127	511910–512059	50	+	putative small toxic membrane polypeptide
BIO00144	583036–582926	37	−	putative outer membrane lipoprotein, cyd operon protein
BIO00301^a^	1056382–1056486	35	+	hypothetical protein
BIO00533^a^	1577459–1577376	28	−	hypothetical protein
BIO00534^b^	1577818–1577543	92	−	hypothetical protein
BIO00587^a^	1717264–1717148	40	−	hypothetical protein
BIO00620	1809435–1809527	31	+	hypothetical protein
BIO00669	1894482–1894333	51	−	hypothetical protein
BIO00670	1894620–1894501	40	−	hypothetical protein
BIO00790	2213607–2213521	29	−	hypothetical protein
BIO00803	2238453–2238557	35	+	hypothetical protein
BIO00855	2421445–2421317	43	−	hypothetical protein
BIO00864	2469896–2469615	94	−	hypothetical protein
BIO00898	2585789–2585685	35	−	hypothetical protein
BIO00936	2769587–2769432	52	−	predicted membrane protein (regulated by cyaR sRNA)
BIO01076	3201904–3202023	40	+	hypothetical protein
BIO01336	4066446–4066339	36	−	hypothetical protein
VP				
BIO01501b	9285–9443	53	+	hypothetical protein
BIO01567^a^	67854–68126	91	+	hypothetical protein
BIO01585	91670–91422	83	−	hypothetical protein
BIO01587^b^	91991–91860	44	−	putative arylsulfatase regulatory protein
BIO01595^b^	105022–105132	37	+	hypothetical protein
BIO01608	135447–135677	77	+	hypothetical protein
BIO01637	153138–153392	85	+	adhesion protein, fragment
BIO01674	183288–183455	56	+	hypothetical protein
BIO01675	183646–183792	49	+	hypothetical protein

a Newly identified sORFs were not annotated in any genome.

b These sORFs were only annotated in one *E.coli* or *Shigella* strain.

### Identification of operon structures

An operon is a series of genes which is co-transcribed in the same transcription unit. Bacterial genes involved in similar functions are often organized into operon structures. DOOR predictions suggested that there were 962 operons in chromosome of *Sf*301 and 60 in the VP [50]. Table S3 shows that we experimentally validated 557 (57.9%) DOOR operon predictions in the chromosome of *Sf*301 and 46 (76.7%) in the VP. Table S4 shows 40 additional coexpressed gene-pairs that were not predicted by DOOR. For example, DOOR predicted that operon 75143 in *Sf*301 was a three gene operon (SF3763–SF3765), but tiling analysis showed that the operon had four genes (SF3762–SF3765) with the inclusion of SF3762. DOOR predictions for a similar operon in *E.coli* K12 MG1655 matched our result. Table S5 shows predicted operon structures that need to be reanalyzed. Of these, 95 operons contained genes encoding a hypothetical protein. For example, DOOR predicted that operon 74376 in *Sf*301 was a five gene operon (SF0040-SF0044). However, our results indicated that the operon should be divided into two parts. Thus, our experiment data might assist in increasing the accuracy of operon annotation.

## Discussion

We published the first *Shigella* genome (*Sf3*01) in 2002[44]. In our initial annotation, we identified 449 sORFs in the chromosome and 76 in the VP, with ten sRNAs identified based on conservation. Recently, we characterized four novel sORFs by integrating a shotgun proteomics method with oligonucleotide array analysis [51]. Here we report the first comprehensive screen for sRNAs and sORFs in *Shigella,* using a combination of bioinformatics and experimental approaches. This is the first genome-wide expression profile of *S. flexneri* genes, pseudogenes, and noncoding regions, which can be used as a basis for the screening of overlooked genes. Tiling array analysis provided further information on expression patterns in different growth phases.

The first bacterial genome was sequenced in 1995 and approximately 1000 completed microbial genomes are now available in the public database (http://www.ncbi.nlm.nih.gov/genomes/MICROBES/microbial_taxtree.html). Numerous prediction programs have been developed to address the problem of annotation. The main strategies used for genome annotation are mathematical models and algorithm-based computational analysis [52], EST/cDNA sequencing[53] and complete set of protein-encoding ORFs cloning[54]. High throughput next generation sequencing instruments have recently revolutionized genomics and genetics, but genome annotation is not keeping pace with the avalanche of raw sequence data. Many researchers are dedicated to bacterial genome annotation, but serious problems still exist. Many annotated genes found in public database are not protein coding genes, but rather ORFs that occur by chance, whereas many actual genes are missing, including sRNAs and sORFs.

The wealth of genomic sequences now available facilitates comparative sequence analysis, which might potentially identify important sequences that cannot be detected by analysis of individual genomes. Differences in bacteria genomes can reflect processes involved in strain adaptive variation under different natural selection, which can endow them with strain-specific biological traits [55]. Growing evidence suggests that gene acquisition via horizontal gene transfer has played an integral role in the evolution of bacterial genomes, and in the diversification and speciation of enteric bacteria. *Shigella* species have a lifestyle that is markedly different from that of closely related bacteria. It is widely accepted that the critical step for *Shigella* speciation was the acquisition of the ancestral form of VP [56]. The functional VP genome is ∼220 Kbp in size and it is composed of a mosaic of virulence genes, maintenance genes, IS elements, and hypothetical genes. In addition to VP, several pathogenicity islands are known in the chromosome of *Shigella* spp.

Transcriptome analysis, using RNA sequencing and high resolution tiling arrays, is beneficial for improving the annotation of sequenced prokaryotic genomes [57]. Tiling array analysis has proved to be a powerful technology, now widely used in eukaryotes and prokaryotes to study transcriptional complexity and identify noncoding transcripts [12], [31], [58], [59], [60]. Tjaden *et al*. assayed the *E. coli* transcriptome under a range of conditions and identified multiple noncoding transcribed elements, including 5′-UTRs, 3′-UTRs, small RNA molecules, and operons [12]. Kumar *et al*. identified 50 sRNAs in the intergenic regions of the *S. pneumonia* strain TIGR4 using tiling array, of which 36 had no predicted function [31].

A wide range of organisms possess ncRNAs, which have roles in a wide variety of processes, including, chromatin accessibility, activator/repressor binding and function, transcriptional initiation, transcription elongation, RNA processing and modification, messenger RNA stability, and translation [2]. Interest in bacterial sRNAs has been fuelled over the past few decades by the availability of numerous complete bacterial genome sequences, which has led to an explosion in the identification and characterization of sRNAs. However, sRNA identification by comparative genomics analysis is only applicable when sequences of several closely related species are available. Previous systematic screens for sRNAs were mainly conducted with the laboratory strain *E. coli* K12, which led to the identification of ∼80 sRNA genes. We can only find sRNAs shared by pathogenic and nonpathogenic strains by comparisons based on conservation of sRNA sequences and structures. Thus, sRNAs unique to pathogenic strains are excluded.

It is now widely accepted that many sRNAs play central roles in gene expression regulation in response to environmental changes. Previous research shows that some sRNAs directly or indirectly regulate virulence genes, or affect adaptive stress responses that are important for bacterial survival in a host [61]. Several studies indicate that many sRNAs are involved in bacterial pathogenesis, including, RNAIII of *S. aureus* and *CsrBCD* of *V. cholerae*. These sRNAs adapt the expression of virulence genes to stress and metabolic requirements [62]. Padalon-Brauch *et al*. pointed out that genetic islands (foreign DNA segments) encoding sRNA genes play an important role in networks that regulate bacterial adaptation to environmental changes and stress conditions, thereby controlling virulence [63]. However, very little is known about *Shigella* sRNAs. Approximately 60 sRNAs are known, but the function of only two sRNAs (RnaG and RyhB) has been studied in *Shigella*. The *S. flexneri* virulence gene *icsA* is critical for the intra- and inter-cellular spreading of the pathogen. This gene encodes an invasion protein, which induces host actin polymerization at one pole of the cell [64]. RnaG is transcribed in *cis* on the complementary strand of *icsA* and regulates at the transcriptional level [49]. *S. flexneri* requires iron for survival and the genes for iron uptake and homeostasis are regulated by the Fur protein. RyhB expression is repressed by Fur. Oglesby *et al*. showed that the acid sensitivity defect of the *S. flexneri* fur mutant is due to RyhB repression of *ydeP*, which encodes a putative oxidoreductase [65]. Murphy & Payne found that RyhB can repress many virulence genes, including those encoding the type III secretion apparatus, secreted effector proteins, and specific chaperones. This phenomenon occurs via RyhB-dependent repression of the transcriptional activator VirB and iron is implicated as an environmental factor contributing to the complex regulation of *Shigella* virulence determinants [66].

We have identified and validated nine novel sRNAs in *Shigella* by combining sRNA identification with tiled microarray probe correlation analysis, transcriptional terminator prediction, and northern blot analysis, but the function of these sRNAs requires further analysis. We also detected 29 novel sORF candidates in *Sf*301 and BLASTX indicated that most encoded hypothetical proteins. We performed more detailed analysis to elucidate the functions of these translated products. Several sRNAs were annotated in genomes based on bioinformatics predictions, but for the first time our results provide support at the transcriptional level. Identification of operon structures is critical for understanding coordinated regulation of bacterial transcriptome, which means that successful identification of operon structures can assist in the functional annotation of hypothetical genes, because proteins encoded by genes in the same operon often have related functions, or share biological pathways[50]. We found that identification of co-expression patterns by tiling array experiments was helpful in operon prediction.

Our approach for global identification of sRNAs and sORFs is applicable to any sequenced microbial species and will accelerate and refine genome annotation and gene identification. Methods for finding sRNAs and sORFs, including computational prediction and experimental validation, are available and continue to develop, but they still fail to provide complete annotation. Our mapping and initial characterization of sRNAs throughout the *Shigella* genome provides significant impetus to the study of these molecules as potential regulators of virulence in *Shigella* and related pathogens.

## Materials and Methods

### Genome sequences

Sequence data of six *Shigella* strains (including VP) were downloaded from GenBank. Accession numbers for the chromosomes are: CP000034, AE005674, AE014073, CP000266, CP000036, and CP000038. Accession numbers for the VPs are: CP000035, AF386526, CP000037, CP000039, AF348706, and AL391753.

### Bioinformatics screening

Known sRNA sequences were extracted from the sRNAMap and Rfam [46], [67] and subjected to BLAST analysis against all sequences mentioned above. We used multiZ to produce a multiple alignment of six chromosomes and VP sequences which were passed on to the RNAz pipeline, according to the manual (cut-off value, P = 0.9). *Rho*-independent terminators were predicted as previously described in Kingsford *et al* [68]. Putative sRNA sequences, including a 50 base pair upstream region, were used for promoter prediction with the Neural Network Promoter Prediction program (http://www.fruitfly.org/seq_tools/promoter.html). BLASTN searches were performed against the nonredundant nucleotide database of NCBI to determine newly identified sRNA sequence conservation from other genomes. sORFs (25–100 amino acids) were predicted using Glimmer, RBSfinder, and GeneMark.hmm, using default parameters [69], [70], [71]. BLASTX searches were performed against the nonredundant protein database of NCBI, for functional annotation.

### Strain and culture conditions

*Sf*301 was cultured overnight at 37°C on Luria-Bertani (LB) agar containing 0.01% Congo red. A single red colony was inoculated into LB medium, without antibiotics, and grown overnight at 37°C and mixed at 250 rpm. An overnight culture of bacteria was prepared for RNA extraction by diluting 1∶50 in 100 ml of fresh medium with aeration by rotary shaking (250 rpm). Growth (optical density, OD) was monitored at 600 nm using an Ultraspec 2000 spectrophotometer (Pharmacia Biotech, Sweden). Cells were harvested in different conditions, as follows: at 37°C in LB medium, in three different growth phases, i.e., lag (OD600<0.2), log (0.2<OD600<1.0), and stationary (OD600>1.0); at 37°C in LB medium with 0.01% Congo red in the log and stationary phases.

### RNA isolation, cDNA synthesis, and cDNA labeling

Total RNA was isolated using a Promega SV total RNA purification kit, according to the manufacturer's instructions. The concentration and purity of RNA were determined using a NanoDrop ND-1000 Spectrophotometer (Thermo Fisher Scientific, USA). Purity and integrity were confirmed by agarose gel electrophoresis. Contaminating genomic DNA was removed from RNA samples via four 30 min incubations at 37°C with 2 ml of Turbo DNase-free, and DNA removal was verified by PCR. cDNA synthesis and labeling was performed following the direct labelling RNA protocol of the IFR microarray facility (www.ifr.ac.uk/safety/microarrays/protocols). Test samples were fluorescently labeled with Cy5-dCTP (GE Healthcare, USA). Separate labeling reactions were pooled after each respective Cy dye incorporation step and then again divided into aliquots to minimize inconsistencies in probe generation. cDNA was purified with a QIAquick PCR purification kit (Qiagen, Germany), according to the QIAquick spin handbook.

### Chip design, hybridization and data analysis

We used a custom-made tiling array containing 386144 probes of the *Sf*301 genome (NimbleGen Systems, USA) for transcriptomics study. Probes were designed with overlapping probes arranged at 25 bp intervals to represent both DNA strands equally and to be nonbiased toward ORFs and/or intergenic regions. Labeled cDNA samples were individually hybridized to the microarray, according to the NimbleGen standard operating procedure. Competitive hybridization was conducted three times for each sample under each test condition. Microarrays were scanned at a 5 µm resolution using a GenePix 4000B scanner (Axon Instruments, CA, USA). Data were extracted using NimbleScan (NimbleGen Systems, USA). Extracted microarray data were analyzed by using NMPP, a user-customized NimbleGen microarray data processing pipeline [72].

We used signals from 280 nonmatching probes, which did not match any region of the genome intentionally placed on our array, to estimate the background level and determine whether a gene was expressed. A gene was considered expressed if its average expression level was greater than five-fold more than the nonmatching probes. All data produced was MIAME compliant and the raw data has been deposited in the Gene Expression Omnibus (GEO) under accession number GSE22800.

### Reverse transcription-PCR (RT-PCR)

We verified sRNA and sORF candidates using a variation of the reverse transcription-PCR (RT-PCR) procedure. We added a primer complementary to the predicted mRNA and reverse transcriptase. After first-strand cDNA synthesis, the reverse transcriptase was inactivated with heat before we added Taq polymerase, and sRNA-specific primers, and sORF-specific primers. PCR products were analyzed using the Agilent 2100 bioanalyzer (Agilent technologies, USA). We observed PCR products under these conditions only when first strand synthesis was conducted with primers complementary to the predicted mRNA. We used the same RNA in the PCR reaction and a negative control to test for genomic contamination.

### Northern blot hybridization

We performed northern blot analysis to verify that sRNAs were transcribed. A total of 18 candidate sRNAs were tested by northern blotting. Table S6 shows the probes used in northern blot study. Total RNA (20 µg per lane) was separated by electrophoresis in an 8% polyacrylamide gel, containing 8 M Urea, and transferred to a nylon membrane by electroblotting. RNAs were cross-linked to the membrane by exposure to UV light. The membranes were hybridized with gene-specific ^32^P end-labeled oligonucleotides, and hybridization signals were visualized using a PhosphorImager (Molecular Dynamics, USA).

### Operons

Two or more consecutive genes were regarded as part of an operon, if they fulfilled the following criteria: (a) they are expressed and transcribed in same direction, and (b) the intergenic region was identified as a single expressed transcript that overlapped the genes in both directions.

## Supporting Information

Table S1 Summary of confirmed sRNAs in Chromosome and virulence plasmid of *Shigella flexneri*
(XLS)

Table S2 List of regions (including known sRNAs) which were identified as containing possible sRNAs genes in chromosome and VP
(XLS)

Table S3 List of confirmed operon predictions.
(XLS)

Table S4 List of newly identified co-expressed genes.
(XLS)

Table S5 List of operon structures need to be re-predicted
(XLS)

Table S6 Probes used in northern blot study
(XLS)
